# Growers’ perceptions and attitudes towards fungicide resistance extension services

**DOI:** 10.1038/s41598-024-57530-z

**Published:** 2024-03-21

**Authors:** Toto Olita, Michelle Stankovic, Billy Sung, Megan Jones, Mark Gibberd

**Affiliations:** 1 Centre for Crop and Disease Management, Curtin University, Bentley, WA 6102 Australia; 2 Consumer Research Lab, Curtin University, Bentley, WA 6102 Australia

**Keywords:** Human behaviour, Biotic

## Abstract

Agricultural extension services have been successful in promoting knowledge sharing and innovation in agriculture. However, the adoption of new agricultural practices, particularly in integrated pest management, has been slow. Using a case study with a co-designed survey instrument, this research aimed to understand how growers in the Southwest Western Australian Grainbelt access information and resources to manage fungicide resistance. We found that the growers rely on a combination of paid agronomists, government or research institutes, local grower groups, and field days for fungicide resistance information. Growers seek information from trusted experts who can simplify complex research, value easy-to-understand communication and prefer resources tailored to their local context. Additionally, growers valued information regarding new fungicide developments and having access to rapid fungicide resistance diagnostic services. These findings highlight the importance of providing growers with effective agricultural extension services to manage the risk of fungicide resistance.

## Introduction

Barley growers manage crop diseases through selection of adapted germplasm, integrated disease management and a heavy reliance on fungicides, often as a preventative measure to avert disease outbreaks^1^. Fungicides impede the infection, growth and reproduction of the pathogens that cause fungal diseases on crops. However, fungal pathogens can have complex population structures and are prone to mutation. The excessive dependence on a narrow range of active compounds in fungicides or the incorrect application of fungicides can select for fungal mutations that confer resistance to these chemicals. Through the repeated application of the same active compound, the proposition of resistance within the pathogen community increases, potentially rendering the active compound to be less effective in controlling crop diseases^2–4^.

Fungicide resistance is a term used when a previously effective fungicide no longer effectively controls crop diseases even when correctly applied. For example, several studies have reported reduced efficacy of fungicides in treating powdery mildew, with instances ranging from reduced efficacy in the field to complete field failure^5,6^. If left unchecked, the prevalence of fungicide resistance will continue to rise, decreasing the effectiveness of current disease management methods and leading to devastating crop losses^7^.

In a global context, the estimated pre-harvest losses due to crop diseases range from 10 to 23% and an additional 10% to 20% occurring during post-harvest stage^8^. These losses equate to a quantity of food sufficient to provide approximately 600–4200 million people with 2,000 calories every day for an entire year^8^. Food security issues continue to rise as the global demand for food is expected to increase^9^. These challenges are expected to compound in the future due to global population growth and the risks posed by climate change^10–12^. Therefore, the ability to grow food sustainably and efficiently is critical for human survival and the loss of fungicides as a disease control measure can have devastating effects beyond those experienced by primary producers.

To address the issue of fungicide resistance and minimise crop losses, it is necessary to develop innovations and extension services that align with growers’ capacity to implement IPM strategies. While IPM guidelines encourage more sustainable long-term pest management practices^12,13^, the adoption of new agricultural practices that are consistent with IPM best practices is frequently slow despite their potential benefits^14,15^. Previous studies have highlighted the challenges associated with adopting sustainable IPM strategies. These include inconsistent application of IPM strategies, unclear guidelines and the economic viability of IPM strategies^16^. The development of fungicide resistance presents a relatively new and emerging challenge to the industry. While there is a growing body of evidence regarding this issue, the awareness of its economic implications remains limited. Additionally, growers often lack support and find it easier and more cost-effective to manage pests using pesticides, even if they have found other IPM strategies beneficial^17^. Given the importance of disease pressure on the viability of food production, fungicides will likely remain an essential IPM option in the future. The implementation of IPM strategies, including deployment of improved host genetic resistance, will not only focus on disease control but will also be essential for sustaining the efficacy of the available range of active compounds used within fungicides.

Farms make an essential contribution to food security, and so, it is crucial for researchers and government organisations to be able to provide farmers with technology and innovations including extension services which increase and sustain crop yields. However, a significant barrier for growers’ adoption of technology and innovations is a top-down “science-push” approach that focuses on transferring technology from experts to farmers while giving little consideration to the input from local growers^18,19^. A study by Anil et al.^19^ found that this approach led to varying adoption rates of new technologies on farms. Additionally, the study highlighted that growers often express concerns about agricultural research when it is only carried out for scientific purposes. Similarly, if the reliability and relevance of the information to the growers was not given priority, it resulted in a disconnect that impacted the adoption of new farming innovations and other services delivered through extension^20,21^. These findings suggest that researchers may not fully understand the needs and concerns of growers when delivering information.

Advancements in agricultural extension have highlighted the importance of involving local growers in research initiatives and fostering collaborations between research institutions and the industry^18,22,23^. However, more work is needed to assess the effectiveness of current IPM adoption models and the adoption rates of sustainable long-term pest management technologies. Historically, extension services were primarily delivered by the public sector^24,25^. However, trends towards larger commercial farms, market-oriented agricultural policies, and an aging and declining rural population have diminished the need for high levels of government funding^24–26^. Consequently, governments in many industrialised nations, including Australia, have reduced their direct investment in agricultural extension, leading to an increased reliance on the private advisory sector for these services^27–30^. However, complete reliance on private extensions has garnered criticism due to limited accessibility to smaller-scale farms and reduced consideration of environmental and sustainability concerns. As a result, a collaborative approach involving both government and private advisors is now recommended^31,32^. Nevertheless, limited research exists on growers’ perceptions and attitudes towards the most desirable fungicide resistance management resources. Additionally, there is a gap in the literature regarding the types of agricultural extension programs that can effectively support growers in addressing fungicide resistance challenges.

Private advisors, such as agronomists, provide growers with specialist support and expert knowledge^33^. In Australia, more than half of the grower population utilises the services of agronomists, with rates varying across regions, and this trend is expected to grow^20^. Growers report that they prefer to keep their operations simple and this leads them to engage private advisors for more complex processes such as precision agriculture-related services; for instance, field mapping, managing spatial data from paddocks and technical equipment support^20^. Thus, agronomists play an essential role in agricultural extension, as they assist growers in implementing new technologies while ensuring operational simplicity.

The high utilisation of agronomists is also influenced by the endorsement of “fee-for-service” advice from peers (e.g., other growers^34^). In contrast to researchers and government extension agents, independent agronomists often establish stronger relationships with growers, often long-term, through regular farm visits^35^. Furthermore, rather than attempting to persuade farmers to adopt new practices or comply with regulations, agronomists focus on providing practical support, with their advice more likely to align with the interests of the growers^33^. Therefore, an independent agronomist is often regarded as an impartial source of advice^33,36^.

Nonetheless, Ingram’s 2008^33^ study acknowledged the presence of power dynamics during interactions between agronomists and farmers. The study recognised that inflexible and authoritarian approaches could negatively affect knowledge exchange. Conversely, there are instances where agronomists compromise on best practices to avoid losing clients. Thus, it is important to examine the role of agronomists within different contexts, particularly from the growers’ perspective. Given the emerging nature of fungicide resistance as a challenge to barley production, understanding the relationships that barley growers establish with agronomists is essential to effectively communicate new innovations.

Collaborating with grower groups is also an important part of agricultural extension. These groups are independent and self-governing community-based organisations comprising farmers and community members who focus on matters related to farm enterprise. This involves actively participating in research trials, creating tailored agri-business solutions to address local needs, and sharing research and development outcomes with other growers^16,37^. The success of the grower groups can be attributed to the shift away from top-down approaches (such as the scientist-to-farmer model) towards community extension approaches that prioritise growers’ input, promote independent learning and foster active participation^16,19,38–40^.

Anil et al.^19^ conducted semi-structured interviews with growers who were members of grower groups to assess the perceived benefits of membership to belonging to the group. The study revealed that growers consider grower groups to significantly influence their learning around new technologies, subsequently impacting their adoption of innovative agricultural practices. Grower groups were identified as being more effective in conducting experiments at the local level than larger national research centres. Additionally, they were identified as the best platform for information sharing. Specifically, field days were regarded as valuable platforms for sharing and collectively addressing challenges, enabling collaborative problem-solving.

The complexity of farmers’ adoption of new technology and practices extends beyond mere technical comprehension^41^. Rather, the process of innovation and practice adoption involves the consideration of values, goals, and social networks, all of which interact with growers’ decision processes^41–44^. Despite the availability of numerous recommendations to growers, only certain innovations and practices are readily and speedily adopted. As new research outputs are generated their utility for on-farm practice change needs to be assessed and in many instances there is a gap between the utility of the output and the intended practice change. Ideally a consideration of utility, and the options available to increase the utility, of research outputs is undertaken at the commencement of a research program through co-design and industry engagement.

In order to establish the utility of outputs related to fungicide resistance, this study conducted in-depth phone interviews with growers in the Southwest Western Australia Grainbelt. The chosen approach aimed to foster the development of collaborative partnerships between researchers and growers, emphasising the value of trust, mutual respect and shared decision-making^45^. The objective of the study was to evaluate growers’ perspectives regarding the existing resources for managing fungicide resistance, identify which resources they readily access as well as investigate the resources growers would like to access and the reasons behind their preferences. Specifically, this research addresses the following research questions:

- *RQ1* What fungicide resistance management extension services do growers currently access?
- *RQ2* What are the motivating factors for growers to access fungicide resistance extension services?
- *RQ3* What other fungicide resistance extension services would growers like to access in the future, and what are the reasons behind their preferences?

## Methods

### Survey design and participant recruitment

The study uses a case study approach to examine growers’ perceptions and attitudes toward resources related to fungicide resistance management. The survey instrument was co-designed with the industry and incorporated a combination of qualitative and quantitative data collection methods. By adopting this approach, we aim to gain a deeper understanding of growers’ unique experiences with fungicide resistance management, allowing us to uncover nuanced insights into growers’ experiences and perspectives. The study was conducted during the 2019/2020 growing season as part of the “Barley Disease Cohort Project”, a collaborative research initiative involving growers in Southwest Western Australia’s Grainbelt. The initiative aimed to evaluate the prevalence of fungicide resistance in the region by examining diseased barley leaf samples obtained from growers. The participants in the Barley Disease Cohort Project were recruited from the medium to high rainfall zone of the Western Australian grain producing areas. The opportunity for participation was created and then promoted publicly (through various media channels, including social media) and farmers were invited to nominate to participate. All interested nominees were accepted into the project.

The study obtained ethical approval from Curtin University Human Research Ethics Committee (HRE2020-0440) and was performed in accordance with the National Statement on Ethical Conduct in Human Research 2007^46^. Growers and agronomists, who had previously consented to be contacted about their fungicide resistance management, were invited to share information regarding their management practices. Prior to participation, participants were presented with the information statement and the consent form. All participants’ informed consent was obtained prior to participation in the study. The primary methods employed for data collection were in-depth phone interviews and online surveys. In order to maintain consistency, the same set of survey questions completed through self-administered questionnaires was read verbatim to participants who completed phone surveys. No additional information was provided to ensure fairness across both survey methods.

### Ethical approval

The study obtained ethical approval from Curtin University Human Research Ethics Committee (HRE2020-0440) and was performed in accordance with the National Statement on Ethical Conduct in Human Research 2007^46^. All participants’ informed consent was obtained prior to participation in the study.

## Results

**Figure 1:**
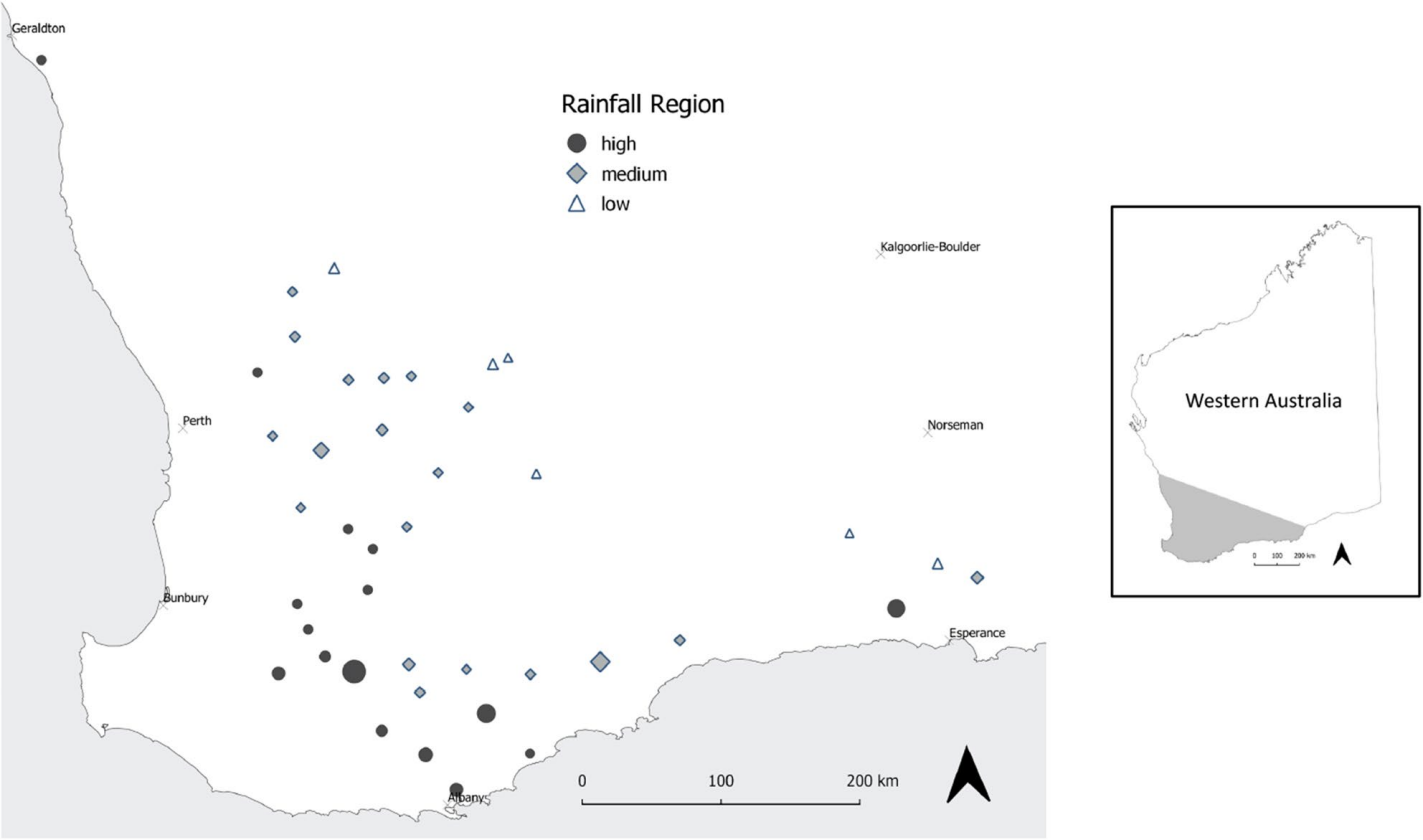
Map of the distribution of survey participants categorised according to postcodes and rainfall zones: Low, Medium and High. The size of the symbols denote the number of participants in each location across the West Australia’s Grainbelt. The map was generated using QGIS software version 3.28.3-Firenze.

A total of one hundred and thirty-seven growers participated in the study, with 82% completing phone interviews and 18% opting for self-administered questionnaires. The age of the participants ranged from 22 to 69 years, with an average age of 44 years. Their experience in the agriculture industry varied from 2 to 54 years, with an average of 25 years. On average, growers had cultivated 1122 hectares of barley across 10 paddocks. The majority of the growers cultivated two barley varieties (48%), with the distribution of varieties ranging from one variety (33%) to five varieties (0.7%). The distribution of the survey participants is shown in Fig. 1, generated using QGIS software version 3.28.3-Firenze^47^.

**Figure 2:**
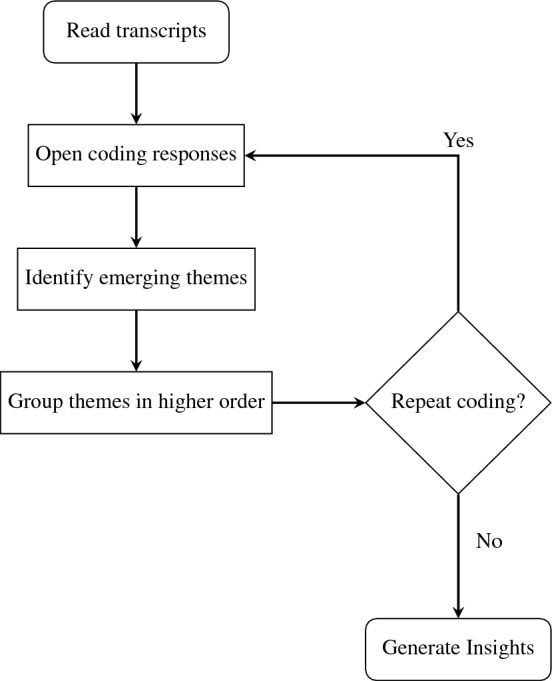
Flowchart illustrating the sequential stages of the thematic analysis.

The qualitative data obtained were manually coded using inductive content analysis, beginning with open coding of the responses^48^. The material was analysed through repeated reading, and any emerging themes were noted to describe all aspects of the content^49–51^. Following a process of abstraction, the identified themes were further grouped under higher-order headings^51,52^. The objective of this systematic analysis, as depicted in Fig. 2, was to generate valuable insights into the underlying factors influencing growers’ preferences for a particular fungicide resistance management resource, thereby shedding light on the decision-making processes involved in disease management. The next section provides a more detailed analysis and discussion of the identified themes.

### Fungicide resistance management extension services

Addressing RQ1, the qualitative data responses (n = 128) show that agronomists are the most utilised resource, with over 84% of growers mentioning them as their primary source of fungicide resistance information (n = 108). Interestingly, agronomists were not only the most commonly mentioned resource but were also the sole source of fungicide resistance information for a significant portion of growers, with over 24% (n = 31) relying solely on agronomists or mentioning them as their exclusive resource. Most growers (i.e., 72% of responses or n = 93) indicated that they generally rely on agronomist advice and reading research or accessing media. Among the preferred sources of fungicide resistance information, electronic and print media from reputable sources were commonly cited. Additionally, growers relied on industry reports, local newsletters, magazines, rural press or did not specify the research sources they accessed. It was common for growers to mention multiple electronic and print media sources, indicating their proactive efforts to acquire and analyse diverse research.

Another significant source of information included discussions and advice from other growers, particularly through interactions with friends and neighbours. For example, *P023: “Communication in the farming sector (friends up north see diseases earlier).”* and *P006: “Friends and neighbours and agronomist”*. Furthermore, growers relied on local agricultural groups (n = 16) such as local farm or grower groups, spray groups and agronomy groups. It was commonly mentioned that these discussions involved individuals from the local area. For example, *P020:“Local farm improvement group with guest speakers.”* and *P031: “We have our local spray group that provides information and this works for me.”*

Field days were also mentioned as another source of information (n = 12), often in combination with agronomist advice, print media and discussions with (local) colleagues. On the other hand, online sources such as Google and Twitter (n = 9), sales representatives and advertising (n = 3) were rarely mentioned. These findings highlight the need for diverse and accessible resources to effectively manage fungicide resistance, taking into account the preferences and reliance of growers on various sources of information and support.

### Growers’ motivations to access extension services

Addressing RQ2, growers were asked about the reasons behind their preferred sources of information related to fungicide resistance management. Through thematic analysis, four key themes emerged that shed light on why growers relied on particular sources of information.

#### Theme 2.1: trusted experts and their connection to research

When accessing industry and government reports, growers considered sources they deemed reliable, trustworthy and current. For example, *P115: “Information that is more relevant, trustworthy, reliable, quality.”* and *P057: “Because the material is fact-checked and substantiated. It’s convenient. Updated material. It’s current and can be accessed in the paddock.”* Growers held the perception that information originating from experts was inherently trustworthy and of higher quality. Agronomists, in particular, were viewed as knowledgeable experts whom growers could confidently rely on to offer reliable and sound advice. One grower stated: *P131: “[My agronomist] is across all the issues, expert in the field, paid service, expect him to give the right advice”* and another *P107: “Available, agronomist is primary because he is knowledgeable, capacity to research.”*

Agronomists were frequently described as reliable, and growers found it easy to place their trust in them. Additionally, agronomists were also seen as the link between growers and cutting-edge research. They were perceived as playing a crucial role in bridging the gap between abstract research, which may seem disconnected from local concerns, with “on-site” or “on-farm” challenges. They carry out research that growers may lack the time or resources to conduct independently, and they also contextualise this research through meaningful conversations. For example, *P010: commented “Agronomist has the final say. They are interface between latest research and grower. Agronomists are knowledgeable as they get paid to know about these issues.”* And *P043: added “Trust the agronomist and the information provided. Respect their opinion. I am glad fungicide resistance management projects are happening - the insights and results from research. Knowledge is power. I don’t have to spend all the money on new chemistry if I don’t have to.”*

#### Theme 2.2: local information is most trustworthy, and you can ‘see’ it in practice

The dispersal of parasitic fungal spores can spread from neighbouring farms or areas through various means, such as wind, rain, and insects. Consequently, local knowledge was flagged as highly important, as this is often the first line of defence against potential challenges related to fungicide resistance management. In one case, the participant *P012: commented “The results that come from [the agronomist] are in the local area. I can relate and get the information out of them easiest.”* One grower offered an additional rationale for relying on the local agronomist, highlighting growers’ preference for experts who are locally accessible and have a proven track record of getting the desired results. For example, *P022: “People lie on social media - pump up the tyres (over-embellish). Trust the people you deal with.”*

Growers valued agronomists’ targeted advice due to their extensive presence and familiarity with the local area. They indicated that agronomists are often the first to identify and understand potential issues before they become apparent on their farms. This enables them to provide targeted recommendations specific to the farm’s needs. Furthermore, agronomists’ frequent visits to the farm further contribute to their ability to offer tailored guidance and support. For example,* P044: “Trust the Agronomist as he is around the whole district and sees the problem before I know about it. The agronomist then is able to provide targeted recommendations. Agronomist has a good understanding of the surrounding area as he has a good spread of clients in similar area that I normally farm.”*

An important aspect frequently discussed within this theme, alongside the significance of local data, was growers’ strong desire to witness technology being tested directly on their own farms. The focus remained on ensuring that the recommendations from experts and research were relevant and tailored to their specific farming context. As grower *P019* pointed out: *“Face to face and in field allows for cross examination of issues with opinions expressed with field data available and visual being made.”* *P120: noted “‘Want the science, see the action, seeing is believing.”* Another indicated *P047: “You can see what’s happening, different rates and applications and all that kind of gaps.”* These findings align with those from *Theme 2.1*, indicating the necessity of translating national or global research practices into actionable recommendations that are guaranteed to be effective on local farms.

#### Theme 2.3: easy and accessible sources of information

The convenience and accessibility of a particular information source were commonly cited as reasons for its usage. Growers noted the importance of having quick and easy access to information, particularly when faced with time constraints and the need for timely information. For example, *P018: emphasised “Convenience. Part of the service agronomist provides.”* *P060: “Time poor. Want to be up to date with the latest literature. Need to have educated conversations with the agronomist.”* *P118: “Easily available in the paddock.”*

Accessibility was also mentioned as a reason for growers to turn to alternative sources of information such as print media. The quotes below illustrate the role of print media as a secondary resource when direct access to agronomists is limited. For example, *P102: “[Print media is] really accessible and agronomists are hard to get hold of...”* and *P035: “Print media is quite selective, I don’t have to troll through web pages. Field days are more like the smallest board of information. Apps are more for identification rather than cures.”*

In each case, sources related to fungicide resistance management were selected based on their capacity to provide targeted information that was readily accessible and easy to understand, enabling practical implementation on farms.

#### Theme 2.4: information communicated through conversation

The importance of exchanging information about disease and fungicide resistance management through conversation was highlighted by many growers. Moreover, growers found these interactions more relatable and easier to understand, particularly in one-on-one settings. For example, they noted the value of face-to-face interactions, which provided them with the opportunity to ask questions and seek specific information relevant to their current crop protection issues. The dynamic and interactive nature of these discussions also enabled growers to report on the progress of their implemented strategies and receive updated responses based on their situation. These quotes provide further illustration:

*P129: “Helped deliver the service to the grower, talking is a better way to learn (prefer to talk), had a mentor all season.”*

*P029: “Your age. Prefer talking to people one on one instead of reading.”*

*P032: “Face to face is better, more relatable, discussing issues you are looking at, one to one interactions are better.”*

Discussion was also highlighted in relation to different sources of information, such as field days, where growers had the opportunity to interact with their peers and gather information about strategies that had proven effective for others in similar situations. For example: *P116: “People can be very easily manipulated, dubious of other information.”* *P093: “Haven’t had exposure to other sources - talking is better, availability.”* Another grower (*P126*) commented: *“The interaction with guest speakers, hands on, knowledge, quickness of feedback.”* In another case, grower *P057* said: *“Because the material is fact checked and substantiated. It’s convenient. Updated material. It’s current and can be accessed in the paddock.”*

Growers also regarded local groups, neighbours and peers as valuable sources of information. These interactions extended beyond mere discussions and were seen as collaborative efforts, enabling growers to work together with their peers to address crop protection issues. For example, *P117* noted: *“Collaborations with other people in the paddock, have robust conversations.”* and *P123* added: *“It’s first hand experience, network, trust in what you see and do.”*

### New extension service

Addressing RQ3 and in order to assess the gaps in fungicide resistance management resources for barley growers, we asked growers which resources they would like to access in the future. Surprisingly, fewer than half of our participants (n = 54) responded to this question, suggesting a possible lack of awareness of alternative options. These findings are consistent with earlier observations, highlighting the reliance of many growers on agronomists as their primary source for accessing the latest crop protection information and technology. Further analysis of the data revealed several emerging themes, which we now explore in more detail.

#### Theme 3.1: more support, information and technology is needed

Despite providing detailed explanations of their resource requirements, the low response rates suggest a sense of uncertainty or limited awareness among growers regarding alternative information sources. Many growers acknowledged the gaps in their knowledge and expressed the need for a better understanding of best practices and region-specific guidelines. Furthermore, there was a strong desire for farm-specific information that they could readily apply on-site. For example, grower *P079* commented: *“Knowing where to turn to. I don’t know where to start to look.”* And another grower (*P120*) said *“More information on what you should do - best practices, tactics on how to deal with fungicide resistance, collaborations.”* Similarly *P130* added *“More information on whether fungicide resistant management practices make a difference on the farm.”*

Growers also expressed the need for additional information, particularly regarding emerging technologies, to be included in the communications they received. In survey responses, growers requested updates on the latest innovations or information about new products as soon as they become available. Moreover, some growers proposed a direct communication approach to overcome the issue of limited information access. They recommended methods such as sending emails directly to farmers and providing updates on new products, new varieties and seed dressings. Another suggestion was to send text messages with regular updates. By establishing direct communication channels with growers, they can stay informed, eliminating the need for a lengthy information-seeking process and thus enhancing accessibility. This approach ensures that growers receive timely and relevant information, enabling them to make informed decisions about their farm operations.

#### Theme 3.2: timely and wider fungicide resistance testing within the local area

Many growers shared concerns about the need for accessible fungicide resistance testing services which would allow for timely updates about the fungicide resistance situation within the local area. They emphasised the need for local district early warning system for fungicide resistance in their area. For example, *P006: “We need local district resistance Information, an early warning of resistance in the area.”* *P010: “Timely and when needed: disease diagnosis, fungicide resistance diagnosis and fungicide management recommendations.”* *P030: “Maybe a quick test: turn around in 1-2 weeks.”* *P034: “A quicker process to see if we do have resistance to fungicides.”* *P003: “In season resistance diagnosis with quick turnaround from sample to result. Waiting months for results is a waste of time.”*

Apart from the timeliness of testing, growers expressed a desire to have access to fungicide resistance information about the wider areas surrounding their farms to enhance their preparedness. They were particularly concerned about the spread of pathotypes from neighbouring areas and wanted to be aware of any potential outbreaks. For example, *P046: “More testing. Information in your immediate district—upwind (local information) and what’s happening further up the windside (neighbouring district).”* The lack of area-specific information poses a challenge to early intervention, as growers may only receive critical information when it is too late. Talking about this issue, grower *P123* said: *“We need more realistic map that considers more than just the hot spots (disease resistance development over time) early warning signs—what that means, field versus laboratory.”*

Growers suggested having access to live databases or maps that provide information on different fungicide resistance outbreaks across the state or region. This would enable them to identify fungicide resistance hotspots in their area and take appropriate action. They also emphasised the importance of automating fungicide application and resistance data, allowing for real-time updates and extraction of relevant information. Commenting on this grower *P038* said: *“We would like to get access to live database or map to view different fungicide resistance around the state or area. To see where fungicide resistance hot spots are so that growers can identify fungicides that are affected in their area and take appropriate action. Automate fungicide application and fungicide resistance data in a live database and able to extract information on the fly- as information is collected it is updated in the database. Collaboration with different stakeholders. Transform manual data collection and manual data entry into automated data to make process much quicker and easier.”*

#### Theme 3.3: easy to understand fungicide resistance test results

Another significant theme concerns the need for easy to understand fungicide resistance diagnoses and results. Growers indicated that the results of fungicide resistance tests (provided through research activities) were often complex and difficult to understand. This aligns with the results from RQ2 where growers identified that they preferred resources that were easy to understand and accessible (*easy and accessible sources*), clear communication (*information communicated through conversation*) and receiving insights from trusted sources (*trusted experts and their connection to research*). Growers consistently faced difficulties translating abstract information into actionable steps that could effectively mitigate disease and fungicide resistance threats on their farms. Suggestions were made to simplify and make the available information more user-friendly, ensuring that growers can easily comprehend and utilise the information provided. In one case, one grower (*P016*) said: *“Making the information that is available more user friendly. Simplifying the information.”* And *P125* noted: *“We need quick turn around test service, confidence in results and fungicide diagnosis that are easy to understand.”* One grower (*P092*) offered an accessible option for communicating information, *“One-stop shop which has everything on one site (e.g., app or website), agglomeration of data.”*

The findings indicate a level of readiness in the industry to engage with commercial fungicide resistance testing or diagnostic services and that such services need to meet the criteria of being accessible, easy to understand and timely. This is likely to become an important guide as research outputs and fungicide resistance tests potentially become an affordable commercial reality.

## Discussion and conclusion

The present research aimed to investigate growers’ perceptions and attitudes towards extension services related to fungicide resistance management. We utilised a qualitative case study approach to capture more nuanced insights into growers’ experiences and perspectives. As the risks associated with fungicide resistance and crop losses continue to increase^5^, it is imperative to understand how growers access information and identify the most effective channels for disseminating information, particularly during periods of high disease epidemics.

We asked growers which extension services and resources they accessed to obtain information related to fungicide resistance management, with a particular focus on the preferred agricultural extension channels. The results reveal that most growers sought guidance from paid agronomists, often in combination with information sourced from government or research institutes. These findings align with prior studies highlighting the widespread preference for private agricultural extensions, with growers valuing the expertise of paid agricultural consultants^53,54^. Our study also found that a considerable number of growers actively participate in networking forums, such as local grower groups and organised field days. These networks also included both government and private research institutes. These findings align with existing research indicating the importance of community extension approaches^19,37,38^. These approaches foster collaborations between public and private entities, making it easier for growers to access relevant information.

We also investigated the reasons why growers prefer to access certain resources, aiming to identify the factors that make certain resources more attractive to them. Growers indicated the need to access trusted experts with connections to research (*Theme 2.1*), which strongly correlated with the use of agronomist services. Specifically, growers noted that hiring an agronomist allowed them to access complex and cutting-edge research without investing significant time, thus overcoming limitations such as time constraints or the lack of training and familiarity with particular methods. These findings are in line with previous research suggesting that growers often rely on agronomists to simplify complex processes^20^.

Another important theme that emerged was the need for local information (*Theme 2.2*). Specifically, growers stated that the agronomist could ‘translate’ research by identifying its applicability to the grower’s current situation and applying it to a local context. Local information was perceived as more trustworthy as growers often expressed the need to witness practical implementation before adopting the technology. These findings suggest that community extension approaches which emphasise grower observation and participation are more likely to lead to the adoption of new technologies^16,19,38,39^. Growers displayed a distrust towards information that lacked clear evidence of its efficacy on their farm, which is consistent with previous work^19,38^.

Regarding the suitability of resources, growers tended to favour resources that were easy to understand and accessible (*Theme 2.3*). They also indicated their preference for information obtained through conversations (*Theme 2.4*). Our findings align with previous work demonstrating the importance of social factors such as communication and teaching for growers to adopt extension services and innovative practices^55^. Moreover, these findings reinforce the importance of promoting community extension approaches that prioritise collaboration and discussion.

To explore missing resources, we identified four additional themes. Growers overwhelmingly expressed the need for more support, particularly in relation to new fungicide developments (*Theme 3.1*). While existing research indicates the availability of crop protection resources, our findings indicate a need for more targeted resources. Previous studies have primarily focused on understanding the reasons behind growers’ hesitancy in adopting new innovations^17,20,21^. While this research is valuable for developing strategies to enhance adoption, our research reveals the importance of actively engaging growers to ensure that research and extension programs effectively address their concerns and meet their specific needs.

Our research found that due to the spread of fungal spores from neighbouring farms through various means such as wind, rain, and insects, two important needs have emerged. Firstly, growers expressed the resounding need for timely commercial fungicide resistance tests and diagnoses (*Theme 3.2*). Secondly, there is a need for wider testing within the local area (*Theme 3.2*). Growers expressed concerns about their lack of preparedness in responding to emerging threats posed by fungicide resistance. They often felt disadvantaged and unable to adequately prepare for new infections due to the scarcity of resources dedicated to addressing fungicide resistance challenges. Consequently, they were often in a reactive position rather than being proactive.

Lastly, growers expressed the desire for easy to understand fungicide resistance test results (*Theme 3.3*). It is evident that growers seek cutting-edge information which is delivered in an accessible way, accompanied by practical guidance on how to action this information. These findings highlight the need to ensure that research findings are accessible and that these inform future commercial testing (should they become available). Ultimately, for any research or innovations to be adopted, it is imperative that the information provided is unambiguous and that there is a clear assessment of the potential benefits that can be derived from their implementation.

The findings of this study have important implications for agricultural extension as they highlight the importance of supporting initiatives that facilitate effective communication between agronomists/advisors and growers. In order to promote the adoption of fungicide resistance management solutions among growers, policy makers should actively support the creation of easily accessible resources and the delivery of local information. For example, within the Australian context, the recommendations by the Australian Fungicide Resistance Extension Network^7^ should focus on the needs of Australian growers and be tailored to Australian growing conditions, along with recommendations from the Fungicide Resistance Action Committee^56^. This can be achieved by encouraging community extension approaches involving agronomists who can translate research into practical applications tailored to the local context. Our findings indicate that agronomists play a significant role in bridging the gap between innovation and practical implementation. Similarly, growers indicated readiness to engage with a commercial testing service with a focus on rapid sample processing and alignment of results with the timing of seasonal activities targeting effective disease management.

Additionally, policies should incorporate proactive measures, such as investments in research, to enable methodologies for fungicide resistance testing and support of research activities focused on fungicide stewardship and industry responses to resistance development. Policies should also facilitate access to timely fungicide resistance testing services. Collectively, these policies would empower growers to address emerging fungicide resistance threats. Equally important is the need for increased investments in research translation communication, which involves clear communication of fungicide resistance test results to growers and providing practical guidance for implementing recommended solutions. This ensures growers receive useful information, leading to greater adoption of extension services.

While our research aligns with previous studies, particularly regarding participation in grower groups, it is important to acknowledge the possibility of a biased sample. Our recruitment was focused on individuals involved in an extension program, which could result in an over-representation of growers who engage with grower groups and research institutes. To overcome this limitation, future studies should target growers who do not have access to grower groups and who have not collaborated with government organisations or research institutes. Moreover, assessing the accessibility and usability of various information sources, particularly for growers with diverse technological literacy levels, is crucial for inclusive communication strategies. Further investigations into the socio-economic factors influencing growers’ information preferences can contribute to targeted interventions. Longitudinal studies tracking the adoption of recommended practices following information dissemination could provide a comprehensive understanding of the practical impact of communication strategies on growers’ disease management decision-making. Addressing these gaps will enhance our understanding of growers’ information-seeking behaviour and inform the development of more tailored and effective communication strategies for sustainable agricultural practices.

It is worth noting that the growers in our sample had an average industry experience of 24.6 years. While we captured a wide range of experience levels, ranging from 2 to 54 years in the industry, it is likely that we have over-represented experienced growers. Therefore, these findings may not be generalisable to inexperienced growers. Notably, a study by Nettle et al.^34^ found that inexperienced growers and those with smaller farms are less likely to access agronomists. Future research should investigate smaller farms and inexperienced growers to ensure their inclusion in agricultural extension programs, particularly in relation to fungicide resistance and other sustainability issues.

## Data Availability

The datasets used and/or analysed during this study will be made publicly available upon the publication of the manuscript.
